# Deciphering Cis-Regulatory Element Mediated Combinatorial Regulation in Rice under Blast Infected Condition

**DOI:** 10.1371/journal.pone.0137295

**Published:** 2015-09-01

**Authors:** Arindam Deb, Sudip Kundu

**Affiliations:** 1 Department of Biophysics Molecular Biology and Bioinformatics, University of Calcutta, Kolkata, West Bengal, India; 2 Center of Excellence in Systems Biology and Biomedical Engineering (TEQIP Phase II), University of Calcutta, Kolkata, West Bengal, India; University of Nebraska-Lincoln, UNITED STATES

## Abstract

Combinations of cis-regulatory elements (CREs) present at the promoters facilitate the binding of several transcription factors (TFs), thereby altering the consequent gene expressions. Due to the eminent complexity of the regulatory mechanism, the combinatorics of CRE-mediated transcriptional regulation has been elusive. In this work, we have developed a new methodology that quantifies the co-occurrence tendencies of CREs present in a set of promoter sequences; these co-occurrence scores are filtered in three consecutive steps to test their statistical significance; and the significantly co-occurring CRE pairs are presented as networks. These networks of co-occurring CREs are further transformed to derive higher order of regulatory combinatorics. We have further applied this methodology on the differentially up-regulated gene-sets of rice tissues under fungal (*Magnaporthe*) infected conditions to demonstrate how it helps to understand the CRE-mediated combinatorial gene regulation. Our analysis includes a wide spectrum of biologically important results. The CRE pairs having a strong tendency to co-occur often exhibit very similar joint distribution patterns at the promoters of rice. We couple the network approach with experimental results of plant gene regulation and defense mechanisms and find evidences of auto and cross regulation among TF families, cross-talk among multiple hormone signaling pathways, similarities and dissimilarities in regulatory combinatorics between different tissues, etc. Our analyses have pointed a highly distributed nature of the combinatorial gene regulation facilitating an efficient alteration in response to fungal attack. All together, our proposed methodology could be an important approach in understanding the combinatorial gene regulation. It can be further applied to unravel the tissue and/or condition specific combinatorial gene regulation in other eukaryotic systems with the availability of annotated genomic sequences and suitable experimental data.

## Introduction

Gene regulation is an essential cellular process that controls the expression level of a gene and thereby controls the phenotypic attributes of an organism. Transcriptional control plays a key role in gene regulation [1–3]. The molecular basis of this control is as following: transcription factors (TFs) bind to their corresponding cis-regulatory elements (CREs), located at the promoters (upstream regions) of target genes and regulate the transcriptional phenomenon. Previous studies established that combinatorial control of gene expression involves association of multiple CREs (and their TFs) at the promoters by combinatorial logic among them [2, 4–6]. Such combinatorial regulation can produce a variety of gene expression patterns which are sometimes tissue and/or condition specific [4, 5].

Several computational approaches intended to understand the CRE mediated combinatorial regulation at the promoter regions of genes [7–11]. If two CREs show a higher tendency to co-occur in a set of promoters, there is a strong possibility that their corresponding TFs regulate the expression of those genes in a co-associative fashion [12, 13]. Sudarsanam et al. [8] used cumulative hypergeometric distribution to identify the co-occurring cis-regulatory elements in the yeast genome. Hannenhalli et al. [13] measured the co-occurrence of cis-regulatory elements by the ‘co-localization index’ to estimate synergistically acting TFs. Pati et al. [14] and Chang et al. [15] applied *Apriori* algorithm to identify the co-occurrence of cis-regulatory elements. In recent years, Vandenbon et al. [16] proposed a novel measuring technique to estimate unbiased co-occurrence tendencies of cis-regulatory elements based on their frequencies of occurrences at the promoter regions. In addition, previous studies showed that combinatorial transcriptional regulation can be analyzed in the form of co-regulatory network [17]. Aiming to further advancement, we integrated the measurement of CRE co-occurrence and network approach that mimics the combinatorics of transcriptional regulation. Here, we introduced a new mathematical expression to compute the co-occurrence tendencies of CREs using their frequencies of occurrences which allowed us to directly transform these binary relationships into a network.

Over the years, co-associative roles of CREs have been analyzed in model plant systems such as Rice [15, 18]. Though, previous attempts have been undertaken to explore the transcriptional regulatory mechanism of gene expression under abiotic stress conditions [18, 19], little such analysis has been done on biotic stresses. Therefore, as a practical application of our computational approach, we attempted to explore the plausible combinatorics of transcriptional regulation of rice under biotic stress condition. For this analysis, we have chosen a specific biotic stress condition, in which rice plants (leaf and root) were infected by a fungal pathogen *Magnaporthe oryzae*. The fungus *Magnaporthe oryzae* is responsible for the blast disease, causing the biggest disruption of annual rice yield (up to 30%) worldwide [20].

Our aim was to establish a new methodology to estimate the co-occurrence relationships among CREs and transform these relationships into networks; this approach made a systems level analysis of the regulatory mechanism possible. Results of this work allowed us to map the CRE-mediated combinatorial regulation of differentially up-regulated rice genes under blast infected condition. Here, constructing the CRE co-occurrence networks, we pointed the probable regulatory cross-talks among different hormone signaling pathways. Further, a network transformation procedure was used to generate a network (clique-clique network) representing even higher order of combinations. The analysis of network topological features suggested that alteration in the CRE combinations is highly facilitated under blast infected condition. Although applied to the blast infected condition of rice, it is a general methodology that can be applied to other experimental studies as well.

## Materials and Methods

**Fig 1:**
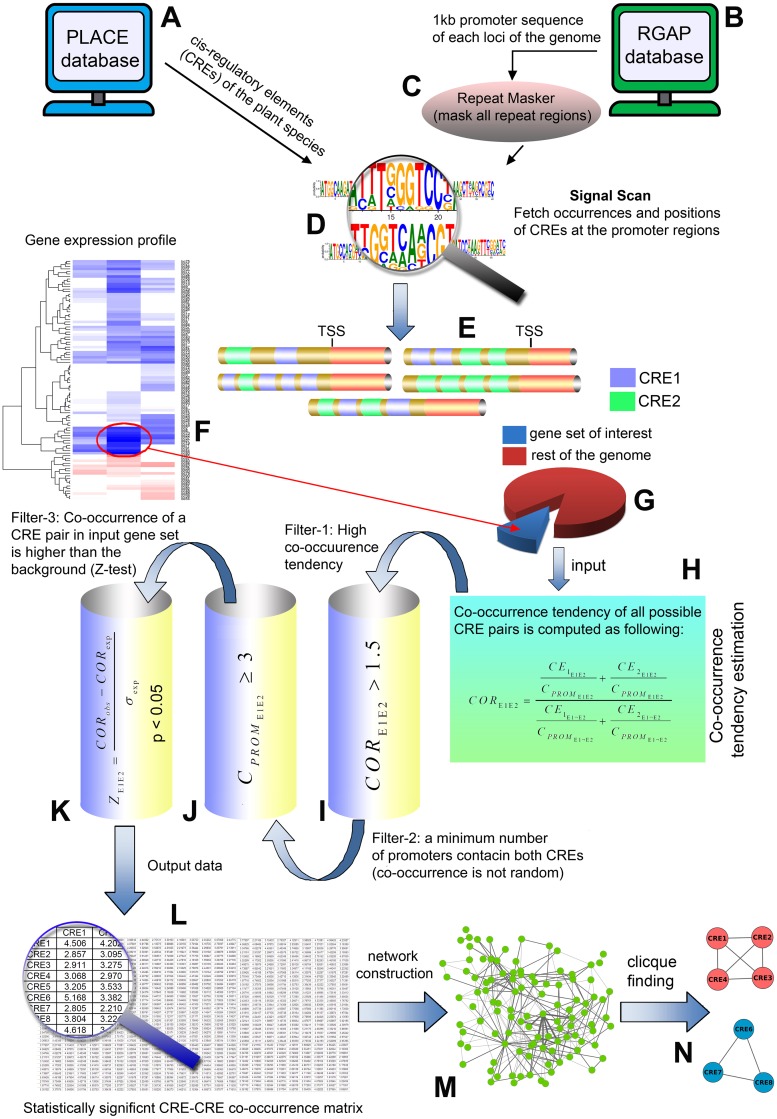
A diagrammatic representation of our methodology. **(A)** Cis-regulatory element information is collected from PLACE database. **(B)** Promoter sequence data is collected from Rice Genome Annotation Project database. **(C)** RepeatMasker masks all the repeated regions in the promoter sequences. **(D)** Signal Scan tool scans the promoter sequences to estimate the occurrences and positions of the CREs. **(E)** A cartoon diagram shows the location of individual CREs in the promoter regions. **(F, G)** Selection of input gene-sets (differentially up-regulated genes) and background (rest of the genome). **(H)** *COR* values are estimated for all possible CRE pairs. **(I)** First filtering step: *COR* > 1.5 values are chosen. **(J)** Second filtering step: only the CRE pairs present in ≥ 3 promoters are selected for further analyses. **(K)** Third filtering step: significance of a *COR* value in the input gene-set is compared against the background by a Z-statistics (*p* < 0.05 is chosen). **(L)** Matrix representation of CRE pairs with statistically significant co-occurrence. **(M)** This matrix is transformed into an edge-weighted network (nodes represent individual CREs, edge weights represent the *COR* values). **(N)** Network analysis reveals the unique cliques of CREs.

In the present work, we introduced a new methodology to identify significantly co-occurring CREs at the promoters of a gene-set. These co-occurrence relationships were further transformed into an edge-weighted network from which higher order combinatorics were estimated. A basic outline of our methodology is presented in Fig 1 and detailed descriptions are given below.

### Data Collection

#### Promoter sequence data

We collected 1 kb upstream promoter sequence data (starting from TSS) [18, 21] of each rice loci from Rice Genome Annotation Project, version 6.1 [22]. RepeatMasker, version open-4.0.2 [23] was used to mask interspersed and simple repeats in the sequence data; this screening prevents unwanted discovery of CREs into the stretches of repeated DNAs present in the promoters.

#### Cis-regulatory element data

An initial dataset of 469 CREs was collected from PLACE (Plant cis-acting regulatory element) database [24]. This initial dataset was screened to identify the unique entries. The final dataset contained 465 CREs with unique consensus sequences. Signal Scan [25] tool was used to fetch the occurrences and positions of the CREs in the promoters.

#### Expression data of blast infected condition

Raw microarray expression data were obtained from Gene Expression Omnibus(GEO) [26], platform accession number GPL2025, for Rice (Oryza sativa) infected with different strains of *Magnaporthe oryzae* (GSE7256, GSE18361) [27, 28]. This platform was designed on Affymetrix GeneChip Rice Genome Array consisting of 57381 probe sets. Raw data were processed and RMA (Robust Multiarray Average) normalization was performed using R and Bioconductor [29]. Differentially expressed probes were obtained (Log fold change > 2, adjusted P-value by BH < 0.01) for individual time point from each experiment. All probes and their corresponding loci information for rice were extracted from the Affymetrix database [30]. Control probes were eliminated from the data set prior to further processing. Those probes mapped to multiple loci were considered as ambiguous probes [31, 32] and were removed from mapping. However, multiple probes mapped to single locus were considered as redundant probes [31] and only the probe having the extreme (maximum/minimum) expression value was mapped to the locus [33, 34]. Finally we got 29766 unique Rice loci mapped to their corresponding probes. We chose our gene-set of interest from these differentially expressed loci. In-house PERL scripts were used for the mapping process.

#### GO mapping and identifying transcription factors

GO mapping was done using the web-based GO enrichment analysis program available at Rice Oligonucleotide Array Database (http://www.ricearray.org/) [34]. Different biological processes, related to plant defense and stress, were identified containing several differentially up-regulated rice loci. The information about transcription factors of rice (*Oryza sativa subsp. japonica*) was extracted from PlantTFDB database version-3.0 [35]. A total number of 2408 loci of transcription factors, distributed into 56 families, were obtained. However, only 1321 loci of transcription factors had corresponding probe mapping and were considered in this work.

### Measurement of co-occurrence tendency of CREs

#### Co-occurrence estimation

We introduced a new mathematical expression to quantify the co-occurrence tendency of a pair of CREs in the promoters of a set of genes. The degree of co-occurrence between a CRE pair, (referred as *E*1 and *E*2), was measured by a parameter termed as the Co-Occurrence Ratio (*COR*).

$$\begin{array}{c} COR_{E1E2}=\frac{\frac{CE1_{E1E2}}{Cprom_{E1E2}}+\frac{CE2_{E1E2}}{Cprom_{E1E2}}}{\frac{CE1_{E1\neg E2}}{Cprom_{E1\neg E2}}+\frac{CE2_{E2\neg E1}}{Cprom_{E2\neg E1}}} \end{array}$$ (1)

Where, *CE*1_*E*1*E*2_ and *CE*2_*E*1*E*2_ are the count of first (*E*1) and second (*E*2) CREs respectively into those promoters comprising both the CREs. *Cprom*_*E*1*E*2_ is the count of such promoters where both the CREs are present. *CE*1_*E*1¬*E*2_ is the count of first CRE (*E*1) in those promoters lacking second CRE (*E*2). *CE*2_*E*2¬*E*1_ is the count of second CRE (*E*2) in the promoters lacking first CRE (*E*1). *Cprom*_*E*1¬*E*2_ is the number of promoters where first CRE (*E*1) occurs exclusive to the second CRE (*E*2). *Cprom*_*E*2¬*E*1_ is the number of promoters where second CRE (*E*2) occurs exclusive to the first CRE (*E*1).

**Fig 2:**
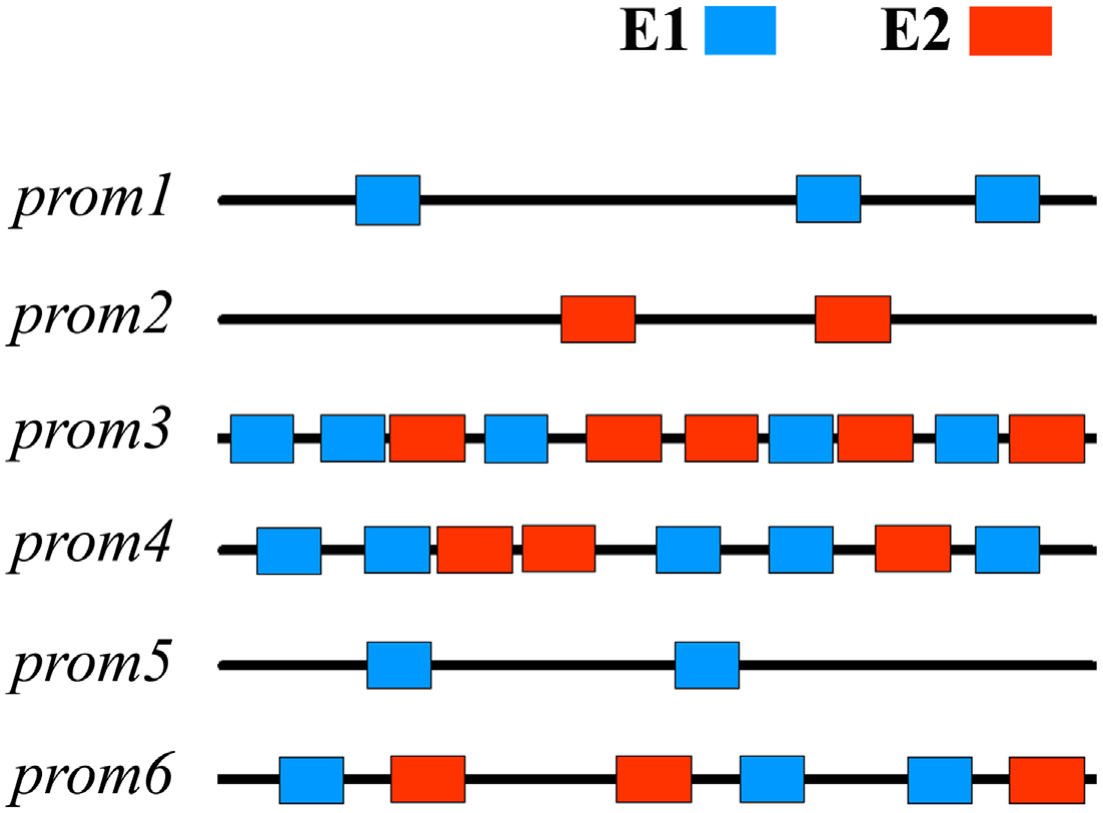
Diagrammatic representation of a pair of CREs (E1, E2) in a set of promoters (*prom*1, *prom*2, … *prom*6). Out of these 6 promoters, 3 contain both the CREs (so, *Cprom*_*E*1*E*2_ = 3), 2 contain the first CRE, *E*1 exclusive to *E*2 (*Cprom*_*E*1¬*E*2_ = 2) and the remaining one contains the second CRE, *E*2 exclusive to *E*1 (*Cprom*_*E*2¬*E*1_ = 1). Similarly, in the first category of promoters *E*1 has occurred 13 times and *E*2 has occurred 11 times (hence, *CE*1_*E*1*E*2_ = 13 and *CE*2_*E*1*E*2_ = 11). The number of occurrences of *E*1 exclusive to *E*2 is 5 (*CE*1_*E*1¬*E*2_ = 5) and the number of occurrences of *E*2 exclusive to *E*1 is 2 (*CE*1_*E*2¬*E*1_ = 2). In this case the estimated *COR* value is 1.77.

The numerator of the above equation (Eq 1) represents the frequency of joint occurrence of a CRE pair (E1 and E2) and the denominator represents the frequencies of exclusive occurrences of these two CREs. Therefore, *COR* value > 1 indicates higher tendency of co-occurrence of two CREs, whereas, *COR* value < 1 means there is a poor co-occurrence tendency. We did not count the occurrences at overlapping sites of the two CREs to restrict the co-occurrence bias emerging from the overlap (i.e *CE*1_*E*1*E*2_ and *CE*2_*E*1*E*2_ were not incremented when their occurrences found at overlapping sites) [16]. We present Fig 2 as an example of the *COR* value calculation. For further details see S1 Text.

#### Setting a stringent cutoff of the COR value

**Fig 3:**
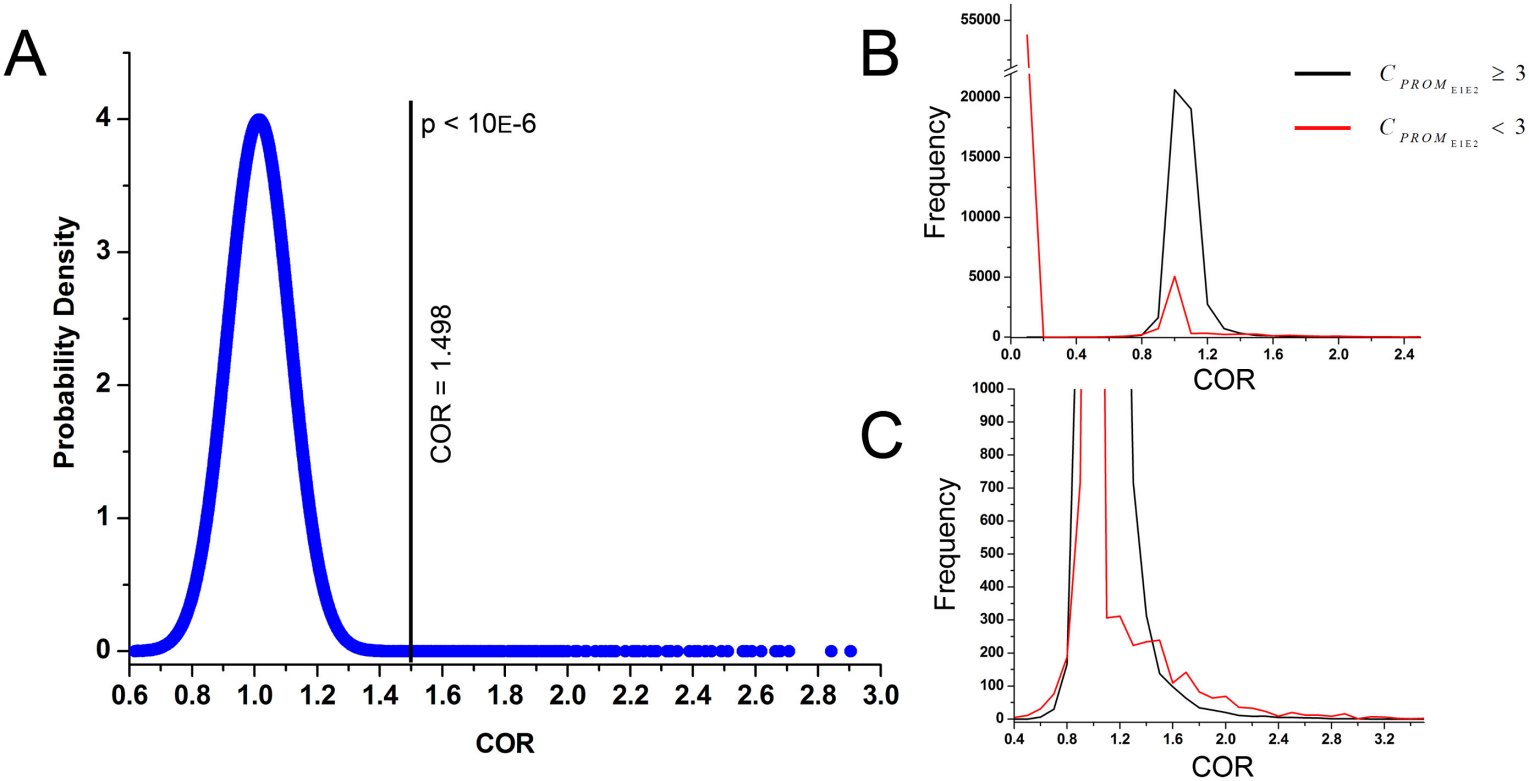
COR value distribution pattern. (A) probability density plot of COR values (B) frequency distribution plot of COR values. (C) magnified view of y axis of the frequency plot of COR values. The red line stands for the COR values of the CRE pairs occurring in < 3 promoters and the black line stands for the COR values of the CRE pairs occurring in ≥ 3 promoters.

We calculated *COR* values for all possible pairs (^465^ *C*_2_) of CREs using the rice promoters. A probability distribution plot of the COR values is provided in Fig 3. The upper-bound confidence interval of the data was computed at 10^−6^ significance level by performing a permutation Z-statistics. In this step, calculation started with the highest *COR* value (observed set) and permuted the remaining values (background) to check whether the difference met the desired significance level. Next, gradually lower *COR* values were added with the observed set and the former step was repeated until it reached 10^−6^ significance level. This enabled us to identify the minimum value of the upper-bound set, which was 1.498. Next, a non-parametric permutation Mann-Whitney U-test (10000 permutation steps) was performed to check whether this upper-bound *COR* values significantly differ from the rest of the dataset. This U-test showed that data above the *COR* value 1.498 was significantly higher than rest of the dataset with a very strong statistical significance (*p* < 10^−170^). Therefore, a *COR* value > 1.5 was considered as the stringent cutoff (S1 Text).

#### Filtering CRE pairs those occur at least in three promoters

A high COR value can also appear from rich occurrence of a CRE pair exclusively in a few (1-2) promoters. Therefore, CRE pairs that do not occur in a considerable number of promoters should be eliminated to minimize the false-positives. The CRE pairs (with *COR* > 1.5) were considered for further analysis only if they were present in at least three promoters (i.e. *Cprom*_*E*1*E*2_ ≥ 3).

#### Estimation of statistical significance of co-occurrence in a gene-set

When analyzing the co-occurrences of CREs within a specific gene-set of interest, it is important to highlight the *COR* values those are statistically significant with respect to the background data. The statistical significance level of co-occurrence of a pair of CREs was determined by another Z-statistics. We checked how well the *COR* value of a CRE pair in an observed promoter set (gene-set of interest) matched the *COR* value that would be expected for random promoter sets of same size. Based on the Z score (Eq 2), one-tailed *p* value was estimated for each pair of CREs.

$$\begin{array}{c} Z_{E1E2}=\frac{COR_{obs}-COR_{exp}}{\sigma_{exp}} \end{array}$$ (2)

[*COR*_*obs*_ → *COR* value found in the actual set of promoters, *COR*_*exp*_ → expected *COR* value which is the mean of the *COR* values calculated from 1000 sets of randomly sampled promoters of the same size of actual set, *σ*_*exp*_ → standard deviation calculated on 1000 expected *COR* values.]

We selected the CRE pairs for which the *p* value of Z-statistics was < 0.05; these pairs were considered for further analysis.

Thus, we applied a set of relevant statistical screening to fetch only the strong co-occurrence signals out of the background data. This statistically significant co-occurring CREs were used for further analysis. The pipeline described here was implemented using in-house PERL scripts.

### Construction of CRE co-occurrence network and clique-clique network

**Fig 4:**
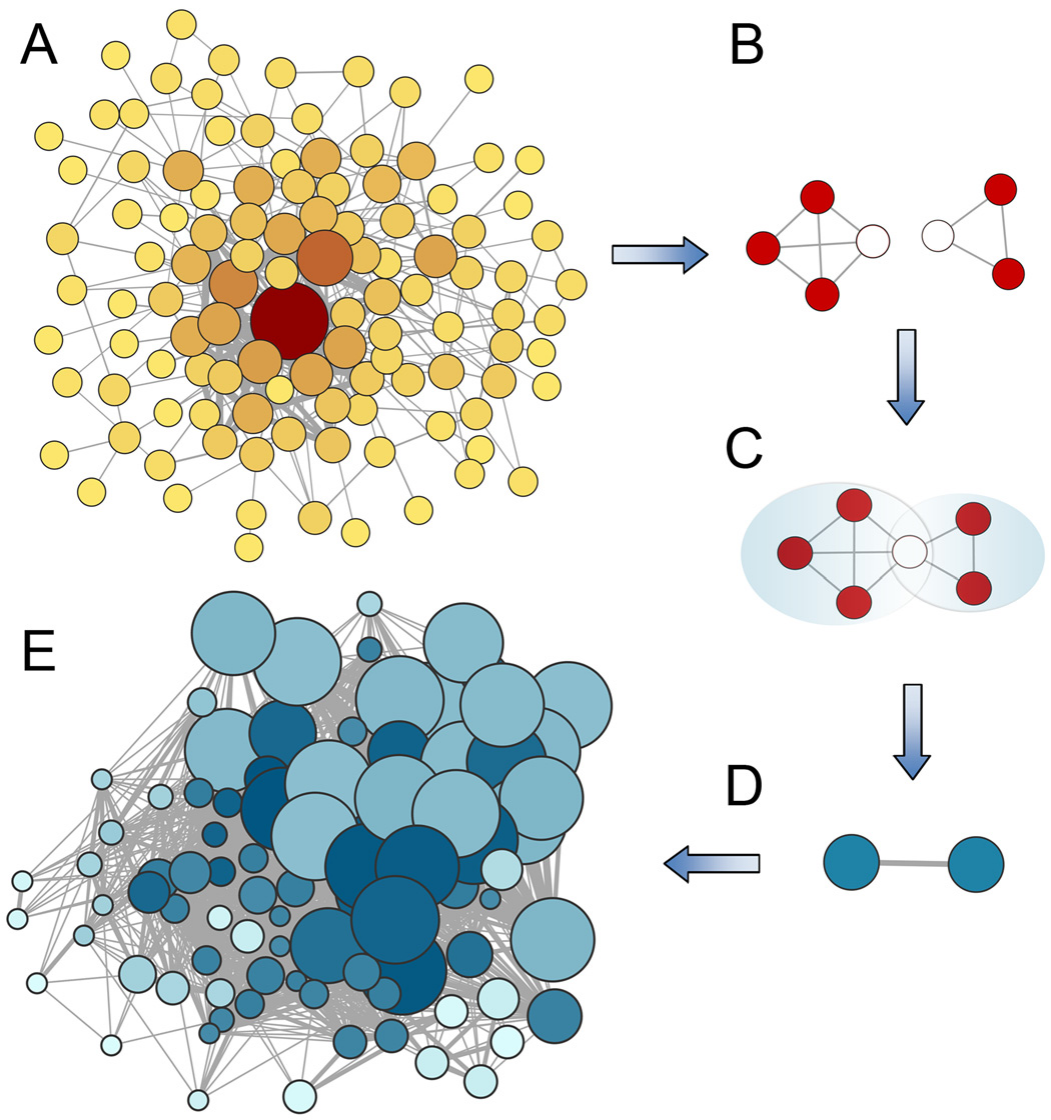
The network transformation. **(A)** A CRE co-occurrences network. In this network, a node represents a CRE and an edge represents co-occurrence relation (*COR*) in between a pair of nodes (Wider line = higher COR value). Node-colour and size represent the degree of a node (darker and bigger = higher degree **(B)** Clique identification in the CRE co-occurrences network. **(C)** Search for common/sharing CRE(s) in between two cliques. **(D)** Represent each clique as single node and connect two cliques(nodes) if common CREs are found in between them. **(E)** A clique-clique network. Here a node represents a clique of CREs, while an edge indicates the relation in between two cliques in the terms of sharing/overlapping of CREs. Wider line of an edge indicates higher number of overlapping CREs in between two cliques. The node-size indicates the number of promoters in which the clique has occurred (bigger = more number of promoters) and the node-colour indicates the degree of a node (darker = higher degree).

For a set of promoters (gene-set of interest), significantly co-occurring CRE pairs were represented as undirected edge-weighted networks, termed as ‘CRE co-occurrence networks’. In these networks, each CRE represented a node and COR values between the CRE pairs represented the edge weights. The CFinder [36] was used to identify the cliques from each network. Clique(*k*) was defined as a complete subgraph with *k* number of nodes. Thus, a clique represents here the combinations of co-occurring CREs in a set of differentially up-regulated genes where their co-occurrences (each CRE co-occurred with every other CRE present into the clique) are statistically enriched. Further, we transformed each CRE co-occurrence network into a clique-clique network where each node represented a clique and an edge weight represented the number of overlapping/sharing CREs between two cliques (Fig 4). Different views of networks, represented here, were constructed using Cytoscape 3.1.1 [37].

### Network topology analysis

We looked into the inherent topology of the CRE co-occurrence networks as well as clique-clique networks. The coefficient of assortativity (*r*) measures the tendency of degree correlation [38]. It is the Pearson correlation coefficient of the degrees at either end of an edge and was defined as:

$$\begin{array}{c} r=\frac{M^{-1}\sum_{i}j_{i}k_{i}-{\left[M^{-1}\sum_{i}0.5\left(j_{i}+k_{i}\right)\right]}^{2}}{M^{-1}\sum_{i}0.5\left(j_{i}^{2}+k_{i}^{2}\right)-{\left[M^{-1}\sum_{i}0.5\left(j_{i}+k_{i}\right)\right]}^{2}} \end{array}$$ (3)

Here, *j*_*i*_ and *k*_*i*_ are the degrees of the nodes at the either ends of the *i* ^*th*^ edge, where, *i* = 1, 2, …, *M*. *M* is the total number of edges. The networks showing positive and negative *r* values are assortative and disassortative in nature respectively.

The clustering coefficient (*C*) is another topological parameter of the network nodes; clustering coefficient (*C*_*i*_) is a ratio *N*_*i*_/*M*_*i*_, where *N*_*i*_ is the number of edges between the neighbors of a *i* ^*th*^ node, and *M*_*i*_ is the maximum possible number of edges between its neighbors. The network clustering coefficient ($\bar{C}$) of a network is computed as the average of all the nodes (*n*) and it represents the cohesiveness of the entire network.

$$\begin{array}{c} \bar{C}=\frac{1}{n}\sum_{i=1}^{n}C_{i} \end{array}$$ (4)

### Measurement of positional distribution patterns of CREs in promoters

We investigated the position-wise distribution patterns of the CRE occurrences. Distribution patterns of their joint as well as exclusive occurrences in rice promoters were studied. Starting from the transcription start site (TSS) we sectioned the 1 kb upstream sequence into 10 intervals, each of 100 nucleotides length. At each interval, the frequency of joint occurrences of the two CREs (when the two CREs E1 and E2 were simultaneously present) as well as their exclusive occurrences (either one of E1 and E2 was present) were computed. At any *i*-th interval the joint occurrence frequency of E1, when it co-occurred with E2 was *F*_*i*_(*E*1*E*2) and the joint occurrence frequency of E2, when it co-occurred with E1 was *F*_*i*_(*E*2*E*1). The Pearson correlation coefficient (*r*) was estimated between *F*_*i*_(*E*1*E*2) and *F*_*i*_(*E*2*E*1) sets (where, *i* = 1, 2, … 10), which measures the similarity of their joint distribution patterns.

## Results and Discussion

### Differentially up-regulated genes in blast infected tissues of rice

Under any biotic/abiotic stress conditions, gene regulation is reprogrammed; for instance, under pathogen attack, the plant defense response is triggered [39]. Defense response involves a change in the entire cellular physiology (induced by remodeling of gene expression by TFs), those try to resist the pathogen in diverse ways.

Cellular functions are activated by elevated rates of expression of the related genes and we have used this elevation as the criterion to select the data sets. Activation of genes related to responsive/signaling pathways should be associated with the elevated rates of expression of the TFs those regulate them [40]. The mode of infection of blast fungus defers in leaf and root, which causes tissue-specific defense response mechanism in rice [28]. In the collected gene expression data, a total number of 302 and 1201 loci were differentially up-regulated at 3 DPI (Days Post Inoculation) and 4 DPI in blast infected rice leaf, respectively. On the other hand 677, 55 and 154 loci were differentially up-regulated at 2 DPI, 4 DPI and 6 DPI in the blast infected rice root, respectively. In leaf, the maximum number of differential gene expression was observed at 4 DPI time point, the stage just before of lesion formation (5 DPI), which indicated a large-scale defense response to hemibiotrophic mode of fungal infection [27]. Where as, in root the maximum number of host gene response was found at 2 DPI time point indicating a substantial and early defense response to biotrophic mode of infection [28]. In addition, Marcel et al. [28] showed that the infection profile of 2 DPI root sample was similar to the two leaf samples (3 DPI and 4 DPI). Though, the 4 DPI time point was common in between leaf and root tissues, in 4 DPI root, the total number of differentially up-regulated genes were only 55. This included only 2 TF genes and few genes related to other GO processes, but excluding those related to defense response. Thus, the 4 DPI root data was found to be insufficient for analysis. Considering all these factors, we selected the input gene-sets from the two expression data sets: 4 DPI of leaf and 2 DPI of root.

In leaf (4 DPI), 112 loci of TFs, belonging to 19 TF families, were up-regulated and in root (2 DPI), 79 loci of TFs, belonging to 12 TF families, were up-regulated out of which 10 TF families (bHLH, bZIP, C2H2, ERF, GRAS, HSF, MYB, NAC, Trihelix and WRKY) were found as common in both tissues. The up-regulated genes from the four TF families (WRKY, ERF, MYB and NAC) were initially chosen for analysis based on the following criteria: (i) the TF families those have well established roles in plants defense response [41–47]; (ii) those were present in higher numbers in both the infected tissues (S1A Fig). In addition, we performed GO functional analysis of the up-regulated rice genes. This identified several GO biological processes, from which we selected four processes based on these two criteria: (i) processes those are known to be involved in plant defense response [27, 48–51] and (ii) those were identified in both the infected tissues. These included “chitin catabolic process” (GO:0006032), “response to stress” (GO:0006950), “defense responses” (GO:0006952) and “G-protein coupled receptor protein signaling pathway” (GO:0007186) (S1B and S1C Fig). We applied our methodology on each of these gene-sets (up-regulated gene-set of 4 TF families and 4 GO processes in infected leaf and root; so total 16 input gene-sets) to fetch the respective CRE co-occurrence networks. Two out of the 16 gene-sets (NAC TF family and “chitin catabolic process” of 2DPI in root) could not be analyzed because the number of loci of each of these sets were insufficient for statistical analyses.

Individual statistics of the co-occurrence networks of each gene-set are provided in the Table 1. The respective lists of input gene-sets, the number of CREs initially found and the number of CRE pairs persisting during the three filtering steps are provided in S1 Table.

**Table 1:** Statistical parameters of CRE co-occurrence networks of the input gene-sets. In the CRE co-occurrence network statistics, *Nodes* refer to the number of CREs present in the network and *Edges* correspond to the number of significantly co-occurring interactions. *Cliques* refers to the number of cliques obtained from each network. *Clique* *size*(*k*) refers to the size of cliques (number of nodes in each clique). All the CREs do not form cliques; here *CREs* *in* *cliques* refers to the number of CREs those appear in cliques.

Input gene-set	Tissue	No. of promoters	CRE co-occurrence network statistics				
			*Nodes*	*Edges*	*Cliques*	*Clique size (k)*	*CREs in cliques*
WRKY	Leaf	20	110	260	38	3, 4	38
	Root	20	113	257	69	3	60
MYB	Leaf	18	112	247	47	3, 4	54
	Root	14	101	212	38	3, 4	47
ERF	Leaf	19	121	347	76	3, 4	97
	Root	23	118	261	44	3	55
NAC	Leaf	15	95	162	17	3, 4	31
	Root	6	—	—	—	—	—
Chitin catabolic process	Leaf	11	93	208	53	3, 4, 5	61
	Root	3	—	—	—	—	—
Response to stress	Leaf	23	122	351	97	3	73
	Root	12	90	210	45	3, 4	61
Defense response	Leaf	19	111	287	88	3, 4, 5	63
	Root	12	83	177	60	3, 4	47
GPCR signaling pathway	Leaf	26	122	291	48	3, 4	57
	Root	11	85	219	59	3, 4, 5, 6	54

### Co-occurrence tendencies and positional distribution of CREs in Rice promoters

We computed the *COR* value of CRE pairs in the promoters of the whole rice genome. A total number of 41335 (38%) *COR* reclined close to 1(0.9—1.1) whereas, 428 (0.39%) CRE pairs were found having *COR* ≥ 1.5 (Fig 3).

**Fig 5:**
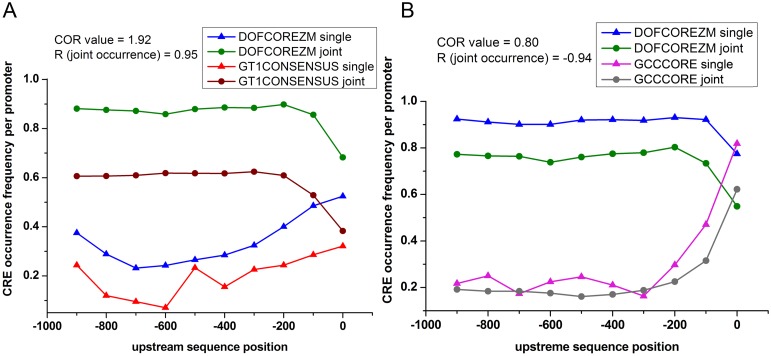
Positional distribution (exclusive/single vs joint occurrence) of CRE pairs. **(A)** The frequencies of occurrence (single and joint) of two CREs (DOFCOREZM and GT1CONSENSUS) with significantly high *COR* value (1.92) is presented. The positional distribution patterns of the two CREs are very similar (*R* = 0.95) where they occur together. Moreover, there is a distinct difference in between frequencies of single and joint occurrences of CREs. The joint occurrence frequencies are about 3 times higher than their corresponding single occurrence frequencies, which indicates the tendency of co-occurrence. **(B)** Positional distribution of DOFCOREZM and GCCCORE exhibit completely different scenario, in which the distribution pattern of single and joint occurrences of each CRE have no difference rather the frequencies of single occurrence are more favored over joint occurrences. As a result, this CRE pair exhibits weak *COR* value (0.80) and highly dissimilar positional distribution pattern (*R* = −0.94) of joint occurrences.

The positional distribution of CREs at promoter region remained one of the important factors to estimate their complex association [52]. Highly similar distribution patterns between the joint occurrences of CREs (e.g., DOFCOREZM and GT1CONSENSUS; *r* = 0.95) were associated with their elevated *COR* values (*COR* = 1.92) (Fig 5). Consequently, dissimilar joint distribution patterns of pairs of CREs (e.g., DOFCOREZM and GCCCORE; *r* = −0.94) were associated with low *COR* values (*COR* = 0.8). In our study, a pair of CREs (GT1CONSENSUS and GATABOX) simultaneously exhibited strong co-occurrence tendency (*COR* = 1.51) and highly similar positional distribution pattern (*r* = 0.94) of their joint occurrences. Earlier, experimental results confirmed a cooperative relationship existing between GT1CONSENSUS and GATABOX [53, 54]. In cellular system, combinatorial control may occur either by the combinatorial logic of CREs [6] or by cooperative interactions of TFs [55]. The *COR* value and the distribution patterns of joint occurrences of two CREs at the promoter regions are likely being the indicators of both the co-regulatory phenomena.

### Regulators being regulated: Auto and cross regulation of TF families

Present study pointed that WRKY and MYB were subjected to auto-regulation [41, 42, 56], since their own CREs were present in their promoters. Many WRKY genes themselves were enriched for W box elements (WRKY71OS, WBOXNTERF3, WBBOXPCWRKY1, WBOXATNPR1 and WBOXNTCHN48) in their promoters, suggesting the possibility of intra-family auto-regulation by themselves. Clique analysis in WRKY promoters revealed that WRKY binding sites, present in their promoters, co-occurred with CREs of other TF families. For example, WRKY71OS, the binding site of rice WRKY71 TF co-occurred with GATABOX (binding site of GATA TF) and MYCCONSENSUSAT (MYC recognition site, bHLH family TF).

Similarly, WBOXNTERF3, another WRKY binding site, formed a combination with ASF1MOTIFCAMV (binding site of ASF-1, a bZIP family TF) and MYBCORE (MYB binding site). This indicated the co-regulation of WRKY genes by a number of TF-families (e.g., WRKY, MYC, GATA, bZIP, MYB etc). Thus, the auto-regulatory loops of WRKY TFs are again associated with complex regulatory cross-talks among different TF-families, some of which play crucial role in plant defense response.

The promoters of MYB family had several binding sites (MYBST1, MYB2CONSENSUSAT, MYBCOREATCYCB1, MYBPLANT, MYB1AT, MYBCORE, MYBPZM and TATCCAOSAMY) of different MYB TFs which indicated the self regulation of this family. A number of cliques of CREs were identified in the MYB promoters pointing the involvement of multiple TF-families in MYB gene regulation. The GT1CONSENSUS (binding site of GT-1, trihelix TF-family) co-occurred with MYCCONSENSUSAT (MYC binding site, bHLH TF-family) and DOFCOREZM (core binding site of DOF TFs). In another clique, ARR1AT (recognized by ARR1 TF) co-occurred with RAV1AAT (recognition site of RAV1 TF, containing AP2-like and B3 domain) and CURECORECR (core sequence ‘GTAC’ of copper responsive element).

The cliques, identified in the promoters of ERF-family genes, contained WRKY binding sites (WRKY71OS, WBOXNTERF3, WBOXATNPR1), bZIP binding sites (ASF1MOTIFCAMV, DPBFCOREDCDC3), MYB binding sites (MYBPZM, TATCCAOSAMY, MYB1AT, MYB2CONSENSUSAT, MYBCOREATCYCB1, MYBGAHV, MYBST1 and EECCRCAH1) and MYC binding sites (MYCCONSENSUSAT, MYCATERD1 and MYCATRD22) indicating a probable cross-regulation by these TF families. In the promoters of NAC family, it was observed that a number of GT-1 binding sites (GT1CONSENSUS, GT1GMSCAM4, GT1CORE) co-occurred with MYC, WRKY, MYB, RAV1, ABRE and GATA binding sites.

All together, our result depicted the complexity of transcriptional regulation by revealing the auto/cross regulations among different TF families. The auto/cross regulation probably performs fine tuning in the expression level of the TF genes and thus may help in the plant defense.

### Involvement of CRE combinations in plant defense suggests cross-talk among multiple hormone signaling pathways

Salicylic acid (SA) and SA-mediated signaling pathways play pivotal roles in systemic acquired resistance (SAR) against fungal infection in plants [57]. Jasmonic acid (JA) mediated signaling pathways are involved with wounding against necrotrophic pathogens [58, 59]. The crosstalk between SA and JA mediated defense pathways involves both synergistic and antagonistic interactions [60, 61]. Here we investigated whether the CRE combinatorics can reflect the essence of these cross-talks among different hormone signaling pathways including SA and JA.

The following clique: [WRKY71OS, MYCCONSENSUSAT, GT1CONSENSUS, DOFCOREZM] was identified in the co-occurrence network of “Defense Responses” genes (S1 Table) differentially up-regulated in leaf. WRKY71OS is the binding site of rice WRKY71 TF, involved in multiple defense response mechanisms such as wounding and pathogen infection, being induced by defense signaling hormones like SA and JA [62]. Moreover, OsWRKY71 was found to be induced by ABA and was repressed in response to Gibberellic acid (GA) in rice aleurone cells [63]. MYCCONSENSUSAT (MYC recognition site) is the binding site of AtMYC2 which is known to be induced by Abscisic acid (ABA) [64] and is involved in Jasmonic acid (JA) mediated defense responses to pathogen attack and wounding [58, 59]. GT1CONSENSUS is the binding site of GT-1 TF (trihelix family) which influences the SA-inducible PR gene expression [65]. DOFCOREZM is the core binding site of DOF, a class of plant specific TF which acts as transcriptional regulator [66]. In this combination the three CREs (WRKY71OS, MYCCONSENSUSAT and GT1CONSENSUS) showed SA, JA and ABA mediated defense activity. Another example is the following clique: [WBOXATNPR1, ARR1AT, MYCCONSENSUSAT, POLLEN1LELAT52], which was identified in the same network. WBOXATNPR1 (another WRKY binding sequence) was identified in the promoters of NPR1 genes of Arabidopsis, has regulatory role in SA-induced defense gene expression and disease resistance. [67]. ARR1AT is the binding site of ARR1 TF, which is regulated by CK [68] and interact with SA to activate plant defense response [69]. POLLEN1LELAT52 was reported as one of the two co-dependent elements involved in pollen specific activation of tomato lat52 gene [70]. While, the two CREs (WBOXATNPR1, ARR1AT) perform SA dependent functions, MYCCONSENSUSAT takes part in JA mediated defense gene regulation [58, 59].

In the co-occurrence networks of “G-protein coupled receptor protein signaling pathway” genes GCCCORE co-occurred with BIHD1OS (in a clique of leaf) and GT1CONSENSUS (in a clique of root). The GCCCORE (GCC box), reported in the promoters of several PR genes, shows JA dependent defense responses interacting with Ethylene response factors(ERF) [71]. The BIHD1OS acts as the binding site of OsBIHD1, a rice BELL homeodomain TF which is known to be activated in rice blast resistance response [72]. While the co-occurrence of GCCCORE and BIHD1OS indicated the JA mediated defense response in rice, the co-occurrence of GCCCORE and GT1CONSENSUS reflected a possible synergism among the JA and SA dependent defense responses.

These CRE combinations indicated a possible scenario in which SA and JA mediated defense signaling may be operated synergistically or antagonistically depending on the requirements of plant immuno-system. The differentially up-regulated genes of these hormone (SA, JA, CK, ABA and ET) biosynthetic processes in infected tissues also suggested their involvement in plant defense response under fungal attack. Finally, this study illustrated the SA and JA mediated transcriptional regulation among defense response genes by multiple TF families and thus, depicting the cross-talk of different hormonal signaling pathways in defense response mechanism.

### Similarities and dissimilarities in CRE combinatorics in blast infected leaf and root

Previously, Marcel et al. [28] established that the biological mode of blast infection is different in leaf and root, and this causes tissue-specific defense response mechanisms of rice. They showed that the gene expression pattern of 2 DPI root sample was similar to the two leaf samples (3 DPI and 4 DPI) and a closer similarity of infection profile was found with 4 DPI leaf. Using these two data sets (4 DPI leaf and 2 DPI root) we investigated whether we can find any similarity/dissimilarity in combinatorial regulation.

**Fig 6:**
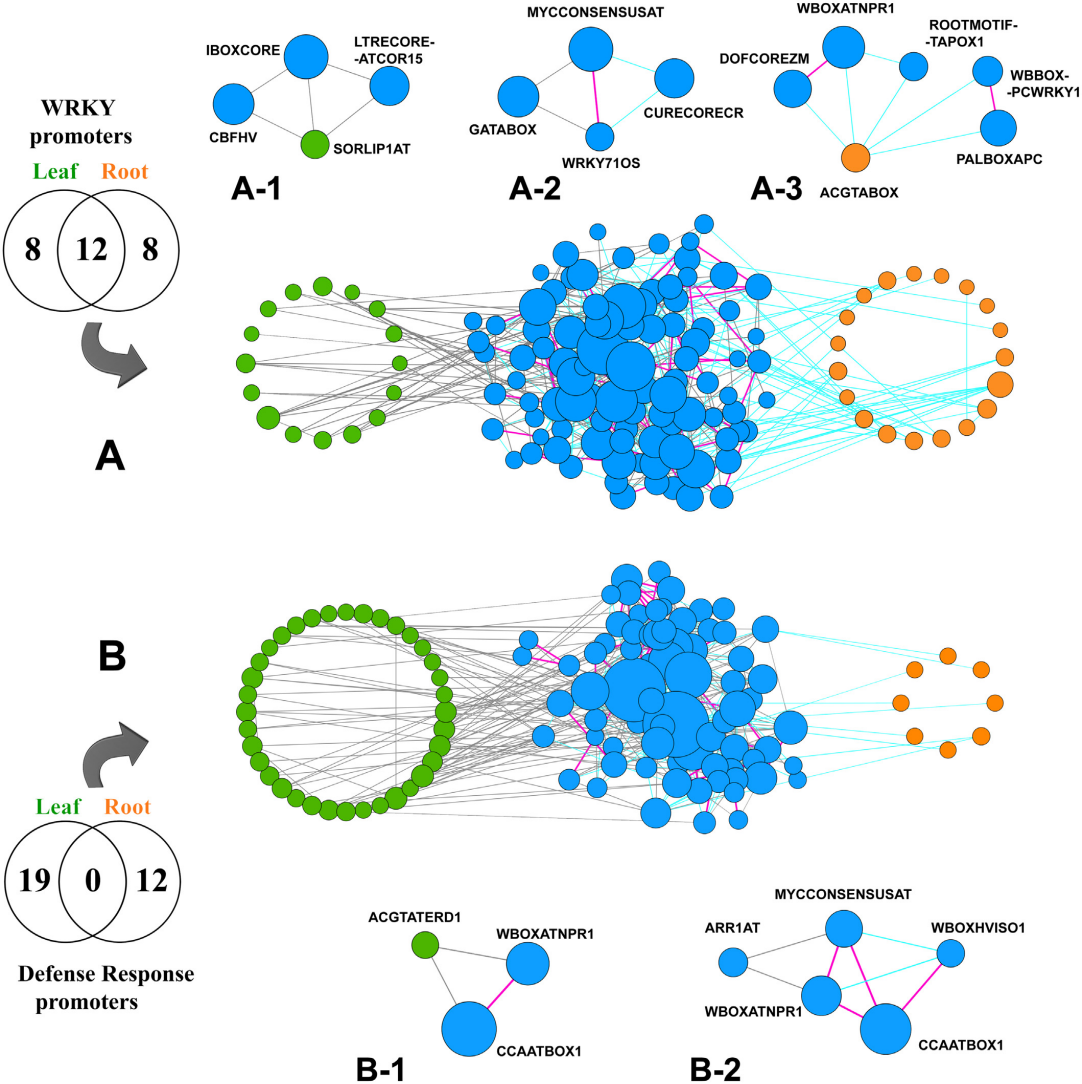
Co-occurrence networks of two pairs of input gene-sets (derived from 4 DPI leaf and 2 DPI root data) and their respective cliques are presented. **(A)** The CRE co-occurrence networks computed for the up-regulated WRKY genes in rice leaf and root tissues are merged into a single one. Nodes (individual CREs) explicitly found in the leaf are highlighted (green) in the left, while those found in the root are highlighted (orange) in the right hand side of the network. Nodes in the middle (blue) are found in both leaf and root tissues. Gray and cyan colour of edges imply that a significant co-occurrence is found between the two connected CREs explicitly in leaf and root respectively. A pink edge implies that the two connected CREs significantly co-occur in both the tissues. **(A-1)** An example of two cliques [SORLIP1AT, CBFHV, IBOXCORE] and [SORLIP1AT, IBOXCORE, LTRECOREATCOR15], explicitly occurred in the network of leaf. **(A-2)** An example of two cliques, found in leaf and root respectively, where they share a common edge (WRKY71OS—MYCCONSENSUSAT). **(A-3)** An example of three cliques [ACGTABOX, DOFCOREZM, WBOXATNPR1], [ACGTABOX, WBOXATNPR1, ROOTMOTIFTAPOX1] and [ACGTABOX, PALBOXAPC, WBBOXPCWRKY1], out of which one clique explicitly occurred in root. **(B)** The merged CRE co-occurrence networks of rice leaf and root, computed for the “Defense Response” gene-sets. **(B-1)** An example of a clique, occurred in the network of leaf. **(B-2)** An example of four cliques, out of which one occurred in the network of leaf, two occurred in the network of root and one is common [CCAATBOX1, WBOXATNPR1, MYCCONSENSUSAT] in both the networks.

We found a number of cliques those exhibit partial differences in between the two data sets. For example, a clique [GATABOX, WRKY71OS, MYCCONSENSUSAT] occurred in the WRKY promoters in leaf and another clique [WRKY71OS, MYCCONSENSUSAT, CURECORECR] was identified in the WRKY promoters in root (Fig 6). While these two cliques share the common CRE pair (WRKY71OS, MYCCONSENSUSAT), the clique in leaf possessed GATABOX (involved in light-response) and the clique in root had CURECORECR (involved in copper and oxygen response) as the non-overlapping nodes. Another example is the following two cliques: [ASF1MOTIFCAMV, MYBCORE, WBOXNTERF3] and [ASF1MOTIFCAMV, MYBCORE, CCAATBOX1]. The first one was identified in leaf promoters and the second one was found in root promoters of WRKY family genes. The biological roles of ASF1MOTIFCAMV, MYBCORE and WBOXNTERF3 were discussed in the previous sections. CCAATBOX1 (binding site of NF-Y) was found in the promoters of HSP (Heta shock protein) genes of tobacco and functioning cooperatively with HSEs (Heat shock elements) to induce the heat-shock activity [73].

The differences were found not only in the cliques but also in the frequecies of the CRE-involvement in the cliques. In the CRE co-occurrence network of “Response to stress”, several MYC recognition sites (MYCCONSENSUSAT, MYCATERD1 and MYCATRD22) were found in different cliques of leaf whereas they were absent in the cliques of root. Significant differences in MYB binding sites and their frequencies of involvement in between the cliques of leaf and root were also observed. We found 9 MYB binding sites (MYBCOREATCYCB1, MYBPLANT, MYB2AT, TATCCAOSAMY, BOXLCOREDCPAL, MYB2CONSENSUSAT and MYBGAHV) in the cliques of leaf, while only 2 of them (MYBST1, MYBPZM) were present in the cliques of root with a significantly lower frequency. In addition, 4 WRKY binding sites (WBOXNTCHN48, WBOXATNPR1, WBOXHVISO1 and WRKY71OS) were involved in different cliques of leaf but only one (WBOXATNPR1) appeared in a single clique of root. This observation showed a clear difference of the co-occurrence tendencies of some TF binding site families (e.g., MYC, MYB and WRKY binding sites) between these two data sets.

Our analysis revealed that in spite of their presence (as nodes) in the co-occurrence networks of both leaf and root, several CREs formed cliques in any one of the tissues. For example, in both (leaf and root) co-occurrence networks of “Defense response” genes, 5 CREs (CCAATBOX1, MYCCONSENSUSAT, WBOXATNPR1, WBOXHVISO1 and ARR1AT) were present as common nodes. But the existing edges (co-occurrence relationship) among these CREs were not common in both the networks. In leaf network, 3 CREs (CCAATBOX1, WBOXATNPR1 and ARR1AT) formed a clique whereas, in root network 4 CREs (CCAATBOX1, WBOXATNPR1, MYCCONSENSUSAT and WBOXHVISO1) were combined in a clique (Fig 6). The clique of 3 CREs (CCAATBOX1, WBOXATNPR1 and MYCCONSENSUSAT) was found as the common clique in both cases.

The CRE co-occurrence networks of up-regulated gene-sets of leaf and root shared some common as well as some unique CREs. These results clearly showed that there exists some similarities as well as some dissimilarities in regulatory combinatorics in between the leaf and root data sets. These dissimilarities might be indicative of the following possibilities: (i) the different biological modes of infection in leaf and root, which induce different defense response patterns, or (ii) the difference of time points (4 DPI in leaf and 2 DPI in root) between the two data sets, or (iii) both.

### Network topology indicates quick transcriptional reprogramming under blast infected condition is highly facilitated

Co-occurrence of CREs at the promoters of a gene implies that TFs are likely acting in a co-associative fashion, involving different TF families. Therefore, a CRE co-occurrence network represents transcriptional co-regulation of a set of genes. On the other hand, a clique (co-occurrence combination) of CREs represents one of the possible higher order combinations of TFs those can regulate the expression of that gene. Thus, a clique-clique network illustrates the different possible higher order regulatory combinatorics (*k* ≥ 3) and the associations among them. We computed two topological parameters of the CRE co-occurrence networks and the clique-clique networks to obtain their inherent properties.

**Fig 7:**
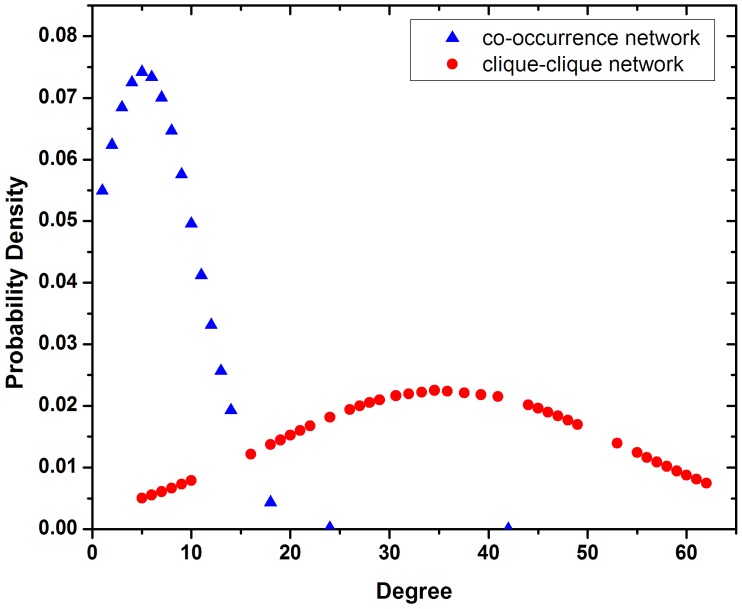
Probability density of degree distribution of CRE co-occurrence network and clique-clique network. The degree distribution pattern shows that there is a better distributed architecture in the clique-clique network compared to the CRE co-occurrence network.

Assortative mixing is a particular network topology which quantifies the tendency of preferential association of the nodes in a network [38, 74] (Eq 3). A network is said to show assortative mixing, if the nodes of similar degree tend to be connected, and disassortative, when nodes of dissimilar degree remain connected. Similarly, the network clustering coefficient ($\bar{C}$) is a global topological parameter which reflects the overall cohesiveness of a network (Eq 4). For each CRE co-occurrence network, we computed the coefficients of assortativity (*r*) and the network clustering coefficients ($\bar{C}$) (Table 2). CRE co-occurrence networks exhibited disassortative mixing and smaller clustering coefficient values. We transformed each CRE co-occurrence network into a clique-clique network (undirected), in which the nodes represent the cliques of CREs and two cliques are connected if they have ≥ 1 sharing CREs. Surprisingly, this transformation entirely changed the inherent topology of the networks. In contrast to the disassortative nature of CRE co-occurrence networks, the clique-clique networks exhibited assortative mixing topology with significantly elevated network clustering coefficient values. For example, the CRE co-occurrence network of MYB TF-family in rice leaf exhibited disassortative topology (*r* = −0.131), while its clique-clique network was assortative (*r* = 0.536). In addition, its network clustering coefficient also elevated from 0.118 to 0.725 (Table 2). Balaji et al. [75] transformed the Yeast transcriptional regulatory network into a co-regulatory network (looking into higher order of combinations); this network transformation revealed the hidden distributed architecture. They observed that at co-regulatory level, a distributed architecture emerges, in which there exists a large number of coordinating partners for any transcription factor. Here, transforming the CRE co-occurrence network into a clique-clique network (represents even higher order of co-regulation) we showed that the later one reflects stronger distributed architecture (Fig 7), supported by high associativity and cohesiveness.

**Table 2:** Network property of CRE co-occurrence networks and clique-clique networks of the input gene-sets. The coefficient of assortativity (*r*) reflects the assortative mixing topology of a network. Negative values of *r* of the CRE co-occurrence networks indicate their disassortative mixing topology whereas, positive values of *r* observed in clique-clique networks imply their assortative mixing topology. Further, the network clustering coefficient ($\bar{C}$) values of the clique-clique networks are significantly higher than their respective CRE co-occurrence networks and this global attribute is an indicator of cohesiveness of the clique-clique networks. Significance is tested by permutation Mann-Whitney U test (No. of permutation = 10000), *p* < 0.001.

Input gene-set	Tissue	CRE co-occurrence network		clique-clique network	
		*r*	$\bar{C}$	*r*	$\bar{C}$
WRKY	Leaf	-0.176	0.052	0.238	0.690
WRKY	Root	-0.176	0.127	0.504	0.684
MYB	Leaf	-0.131	0.118	0.536	0.725
MYB	Root	-0.161	0.134	0.094	0.775
ERF	Leaf	-0.138	0.165	0.433	0.675
ERF	Root	-0.104	0.127	0.439	0.622
NAC	Leaf	-0.007	0.086	0.627	0.435
NAC	Root	—	—	—	—
Defense response	Leaf	-0.116	0.211	0.273	0.828
Defense response	Root	-0.132	0.209	0.112	0.871
Response to stress	Leaf	-0.161	0.133	0.538	0.672
Response to stress	Root	-0.103	0.135	0.325	0.679
Chitin catabolic process	Leaf	-0.033	0.231	0.459	0.723
Chitin catabolic process	Root	—	—	—	—
GPCR signaling pathway	Leaf	-0.07	0.115	0.365	0.781
GPCR signaling pathway	Root	-0.006	0.237	0.313	0.721

In cellular system, an efficient reprogramming of transcriptional regulation is necessary for quick response against external perturbation like pathogen attack. A clique-clique network represents the higher order combinatorics of transcription regulation of a set of genes, the ability of the transcriptional reprogramming should be reflected in its topology. Here, we observed that they simultaneously exhibit strong assortative mixing of nodes as well as higher clustering coefficients. These features illustrate that the CRE combinations, central to the regulation, tend to associate with several other CRE combinations those are also central to the regulation. This might be an indication of the ability of efficient alteration in the combinatorial logic of CREs so that a quick change of transcriptional regulation may occur at the time of blast infection.

## Summary

Here, we proposed a methodology that first quantified the co-occurrence tendencies of CREs in a gene-set of interest, filtered these scores to obtain significantly co-occurring CRE pairs, transformed these pairs into networks and analyzed these networks to fetch higher order CRE combinatorics. This allowed us to perform system level analysis of regulatory phenomena. To demonstrate the potential applicability of our methodology, we applied it to the blast infected condition of rice tissues.

We observed that strong co-occurrence of some CRE pairs are associated with a specific positional distribution pattern of their joint occurrences at the promoter regions. In a few cases, such highly similar positional distribution and simultaneous strong co-occurrence tendency of CREs were associated with the respective TF cooperativity.

Transcriptional control of gene expression involves auto/cross regulation among various TF families. The co-occurring CREs found at the promoters of TF genes indicated auto-regulation and cross regulation. In this study, preparing CRE co-occurrence networks, we showed that the essence of cross-talk among multiple hormone signaling pathways can be captured in the form of cliques of CREs. For example, during blast infection, we found that CREs associated with SA and JA mediated signaling pathways made up several cliques in an intermingled fashion. CRE co-occurrence networks of leaf and root data sets revealed a number of differences as well as similarities of CRE combinatorics in between them.

Social networks exhibit assortative mixing topology which is generally lacking in the non-social networks (e.g., biological networks) [76, 77], with a few exceptions like, amino acid contact networks [78] and co-evolutionary networks within macromolecular complexes [79]. Here, the disassortative CRE co-occurrence networks were transformed into assortative clique-clique networks. This observation clearly unraveled the hidden property of the higher order of combinatorial regulation with an indication that biological networks might exhibit a social network-like behavior.

Transcription regulation and its involvement in altering cellular physiology under various stressed conditions has been proven difficult to resolve due to the inherent complexity of the process. As a result, even after decades of research, a systematic description of *in vivo* transcription regulation remains elusive. In this study, we conveyed a potential methodology to compute co-occurrence tendencies among cis-regulatory elements and its application to reveal the regulatory cross-talk among multiple defense signaling pathways. One can apply this computational approach to explore the combinatorial gene regulation in other eukaryotes, under any cellular condition.

## Supporting Information

S1 Text This file describes the significance of the new metric *COR* value to estimate the co-occurrence tendencies of CREs. The calculation of *COR* value under different possible cases are illustrated. In addition, the significance of *COR* value cutoff 1.5 is shown here.
(PDF)

S1 Fig Differentially up-regulated rice genes under blast infected conditions. **(A)** Differentially up-regulated TFs in infected leaf and root of rice. The X axis represents individual TF-family and the Y axis represents the numbers of differentially up-regulated TF genes from each family. **(B), (C)** The pie charts representing the GO biological processes which mapped to ≥ 10 differentially up-regulated loci of infected rice leaf and root, respectively. In leaf 882 (out of 1201) loci and in root 502 (out of 677) loci are shown here. **(D)** The intersect region of each Venn diagram indicates the number of common up-regulated loci of respective gene-sets in between leaf (4 DPI) and root (2 DPI).
(PNG)

S1 Table Detailed information about the input gene-sets and the results obtained by using the proposed methodology. (XLS)
