# Supplementary material for: Deciphering Cis-Regulatory Element Mediated Combinatorial Regulation in Rice under Blast Infected Condition
Source: PLoS One. 2015 Sep 1;10(9):e0137295. doi: 10.1371/journal.pone.0137295 (PMC4556519; doi:10.1371/journal.pone.0137295)
Supplement: S1 Text — The calculation of COR value under different possible cases are illustrated. In addition, the significance of COR value cutoff 1.5 is shown here. (PDF) [file pone.0137295.s001.pdf]

Assigning a new metric to estimate the co-occurrence tendencies of CREs

Vandenbon et al. [1] proposed a novel computational approach, which estimates the co-occurrence tendency of cis-regulatory elements in an unbiased fashion. They define a co-occurrence score for two CREs A and B in terms of a Frequency Ratio (FR) parameter. This parameter can be computed for CRE A, considering CRE B is present and vice versa, resulting two different $FR(A|B)$ and $FR(B|A)$ values in the two respective cases (see Table 1). In our study, we aim to understand the combinatorics of CRE-mediated gene regulation. For this purpose, we require a methodology which can assign a single co-occurrence score for each pair such that we can transform them into an edge-weighted network. Certainly, the methodology used by Vandenbon et al. [1] fails to incorporate this purpose. The metric we have used assigns one single co-occurrence score (COR) for each CRE-pair and comparing against the background data, it extracts only the statistically significant co-occurrence scores in an unbiased fashion.

In the following, we have presented a descriptive table to show how the metric used by Vandenbon et al. [1] assigns different $FR(A|B)$ and $FR(B|A)$ values and in the same cases, how our metric assigns a single co-occurrence score. This data is generated on the same dataset, the whole rice genome, and only a few CRE pairs are mentioned as an example.

Table 1. Few examples of CRE pairs are presented here along with their $FR(A|B)$ and $FR(B|A)$ values (proposed by Vandenbon et al. [1]),and COR values, proposed in our methodology. For an individual CRE pair, the $FR(A|B)$ often differs from $FR(B|A)$ which restricts the CRE pairs to transform into a network. Whereas, in our calculation, a single value (COR) represents the co-occurrences tendency of a pair of CREs and thus it allows the binary relation to directly transform into a network.

**Motif A**	**Motif B**	$FR(A\|B)$	$FR(B\|A)$	COR_{AB}
ARR1AT	ASF1MOTIFCAMV	0.92	0.61	1.03
ARR1AT	BIHD1OS	0.99	1.26	1.25
ARR1AT	CCAATBOX1	1.16	2.21	1.21
ARR1AT	GT1CONSENSUS	1.53	2.01	1.88
ARR1AT	GATABOX	1.40	2.67	2.06
ARR1AT	MYCCONSENSUSAT	1.02	1.31	1.58
ARR1AT	WRKY71OS	0.91	1.04	1.67
ASF1MOTIFCAMV	GCCCORE	1.37	1.45	1.13
BIHD1OS	DOFCOREZM	1.82	1.04	1.32
CAATBOX1	DOFCOREZM	2.01	1.75	2.62
DOFCOREZM	WRKY71OS	0.96	1.24	1.71
DOFCOREZM	GT1CONSENSUS	1.92	2.56	1.92
DOFCOREZM	GCCCORE	0.80	0.25	0.81
GATABOX	GCCCORE	0.76	0.29	0.80
GATABOX	GT1CONSENSUS	1.85	1.58	1.51
GATABOX	MYCCONSENSUSAT	1.04	1.21	1.50
GATABOX	WRKY71OS	1.12	1.22	1.62

COR value calculation: case study

In our methodology COR value is defined as

$$COR_{E1E2} = \frac{\frac{CE1_{E1E2}}{Cprom_{E1E2}} + \frac{CE2_{E1E2}}{Cprom_{E1E2}}}{\frac{CE1_{E1\neg E2}}{Cprom_{E1\neg E2}} + \frac{CE2_{E2\neg E1}}{Cprom_{E2\neg E1}}} \quad (1)$$

At the time of estimation of COR value (using different input promoter sets) various situations may occur because there are differences in distributions and frequencies of CREs in the genome. These are elaborated in the following cases.

case 1: Frequencies of joint occurrences of E1 and E2 are higher than their frequencies of exclusive occurrences

$prom1$	**E1**	**E2**	**E1**	**E1**	**E2**	E3	...
$prom2$	**E1**	**E2**	**E2**	**E1**	**E1**	**E2**	...
$prom3$	**E1**	**E2**	**E1**	**E2**	**E2**	E4	...
$prom4$	**E1**	**E1**	**E2**	**E1**	**E2**	E3	...
$prom5$	**E1**	**E1**	E3	E3	E4	...	...
$prom6$	**E1**	**E1**	E4	E5	E4	...	...
$prom7$	**E2**	**E2**	E3	E4	E5	...	...

So, $COR_{E1E2} = \frac{\frac{11}{4}+\frac{11}{4}}{\frac{4}{2}+\frac{2}{1}} = 1.37$ (i.e., $COR_{E1E2} > 1$).

case 2: Exclusive occurrence of any one CRE (E1 or E2) is absent in input promoter set

$prom1$	**E1**	**E1**	**E2**	**E1**	**E2**	E3	...
$prom2$	**E1**	**E2**	**E2**	**E1**	**E1**	**E2**	...
$prom3$	**E1**	**E2**	**E1**	**E2**	**E1**	E4	...
$prom4$	**E1**	**E1**	**E2**	**E1**	**E2**	E5	...
$prom5$	**E1**	**E1**	E3	E4	E5	...	...
$prom6$	**E1**	**E1**	E3	E3	E5	...	...
$prom7$	**E1**	**E1**	**E1**	E4	E5	...	...

So, $COR_{E1E2} = \frac{\frac{12}{4}+\frac{11}{4}}{\frac{7}{3}+0} = 2.46$ (i.e., $COR_{E1E2} > 1$).

case 3: No exclusive occurrence of E1 and E2 in input promoter set

$prom1$	**E1**	**E1**	**E2**	**E1**	**E2**	E3	...
$prom2$	**E1**	**E2**	**E2**	**E1**	E4	E5	...
$prom3$	**E1**	**E2**	**E1**	**E2**	**E1**	E4	...
$prom4$	**E1**	**E1**	**E2**	**E2**	**E2**	E5	...
$prom5$	**E1**	**E2**	**E2**	**E1**	E4	E5	...
$prom6$	**E1**	**E1**	**E2**	**E2**	**E1**	E3	...
$prom7$	**E1**	**E2**	**E1**	**E2**	E3	E5	...

$COR_{E1E2} = \frac{17}{7} + \frac{15}{7} = 4.57$ (i.e., $COR_{E1E2} > 1$).

In this case, the numerical value of the denominator is zero, for which the COR value turns out to be infinite. To avoid this case, we pseudocount the denominator as unity, which results a high (expected in this case), but non-infinite COR value.

case 4: No joint occurrences of E1 and E2 in input promoter set

*prom*1	**E1**	**E1**	**E1**	E3	E4	...
*prom*2	**E2**	**E2**	E3	E4	E5	...
*prom*3	**E1**	**E1**	E3	E4	E4	...
*prom*4	**E1**	**E1**	**E1**	E3	E5	...
*prom*5	**E1**	**E1**	E3	E3	E4	...
*prom*6	**E2**	**E2**	**E2**	E5	E4	...
*prom*7	**E2**	**E2**	E3	E4	E5	...

$COR_{E1E2} = \frac{0+0}{\frac{10}{4}+\frac{7}{3}} = 0.$

case 5: Frequencies of exclusive occurrences of E1 and E2 are higher than the frequencies of their joint occurrences

*prom*1	**E1**	**E2**	**E1**	E3	E4	...
*prom*2	**E1**	**E2**	**E2**	E3	E5	...
*prom*3	**E1**	**E2**	E3	E4	E4	...
*prom*4	**E1**	**E1**	**E2**	E3	E5	...
*prom*5	**E1**	**E1**	E3	E3	E4	...
*prom*6	**E1**	**E1**	E4	E5	E4	...
*prom*7	**E2**	**E2**	E3	E4	E5	...

Here, $COR_{E1E2} = \frac{\frac{6}{4}+\frac{5}{4}}{\frac{4}{2}+\frac{2}{1}} = 0.68$ (i.e., $COR_{E1E2} < 1$).

FR calculation (equation 2 proposed by Vandenbon et al. [1]) also yields similar results in this case.

$$FR(B|A) = \frac{\frac{n(B|A)}{seq(A)}}{\frac{n(B|!A)}{seq(!A)}} \qquad (2)$$

considering A = E1 and B = E2,

$FR(E2|E1) = \frac{\frac{5}{4}}{\frac{2}{1}} = 0.625$ (i.e., $FR(E2|E1) < 1$).

and, $FR(E1|E2) = \frac{\frac{6}{4}}{\frac{4}{2}} = 0.75$ (i.e., $FR(E1|E2) < 1$).

Though the two CREs (E1 and E2) are present together in 4 promoters, the COR as well as FR score less than 1. Here, it worths mentioning that only just occurring together in promoters does not confirm strong co-occurrence tendency of two CREs; rather the frequencies of occurrences (joint compared to exclusive) determine the tendency of co-occurrence. A number of studies have confirmed that frequency of occurrences of a CRE at promoter regions is an important factor to predict their corresponding TF activity [1–7]. Simultaneously, higher frequency of occurrences of multiple CREs is a more accurate predictor of the respective cis-regulatory modules [2–4]. Our proposed metric, COR value, takes into account these factors and estimates the co-occurrence tendencies of CREs. Therefore, the above outcomes are as expected and relevant.

The accuracy of a methodology (to estimate something) reflects in its ability to minimize false positives. As stated earlier, higher frequency of occurrence of a pair of CREs is a more accurate predictor of their combinatorial regulation in a set of genes by their respective transcription factors. In "case 5" like situations, we see that the joint occurrence frequency of a CRE pair is less than their exclusive occurrence frequencies, resulting $COR < 1$. Since the COR value is lower than our defined threshold, a statistically significant conclusion cannot be made in such cases. This scenario might be informative to a possible co-associative role, or it might be a false positive as well. So we excluded "case 5" like situations from further analysis.

The significance of COR cutoff 1.5

Here we have generated a scatter plot of the two parameters $[\frac{Cprom_{E1E2}}{Cprom_{E1\neg E2}}]$ and $[\frac{Cprom_{E1E2}}{Cprom_{E2\neg E1}}]$. The first parameter indicates the ratio of the number of promoters ($Cprom_{E1E2}$) where E1 and E2 co-occur and the number of promoters ($Cprom_{E1\neg E2}$) where E1 occurs exclusive to E2. Whereas, the second parameter indicates the ratio of the number of promoters ($Cprom_{E1E2}$) where E1 and E2 co-occur and the number of promoters ($Cprom_{E2\neg E1}$) where E2 occurs exclusive to E1. A diagonal is generated at 45 degree slope and a 10% deviation from this diagonal at both tails of the distribution are considered to define a specific area in 2D space. Any data-point located surrounding this diagonal, indicates that for both the CREs, the number of promoters including their co-occurrences is higher than those including either of their exclusive occurrences. Tendency of being located within this area is computed by randomly picking 100 points from all 6 sets and computing the percentage of them being located within this area. The Z score of significance is computed; p-value of significance is defined as the complementary error function of the Z-score. CRE pairs with $COR > 1.5$, are mostly found around the diagonal; while we reduce the threshold, the tendency of finding an off-diagonal data points drastically increases (see Fig. 1). This observation suggests that COR value > 1.5 is not only the indication of strong co-occurrences of the respective CRE pair but also an indication that the number of promoters having the CRE pair is much more abundant in the genome than those having either one of them. Moreover, when COR value is ≥ 1.5, the abundance of $Cprom_{E1E2}$ is almost equally higher than both $Cprom_{E1\neg E2}$ and $Cprom_{E2\neg E1}$.

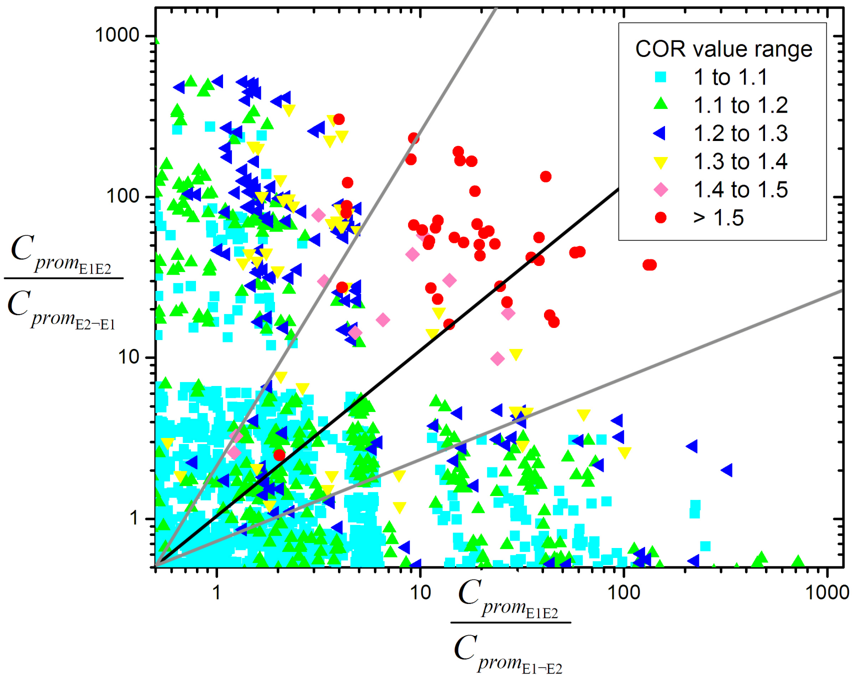

Fig. 1. Scatter plot of different range of COR values. Both the X and Y-axis are in logarithmic scale.

References

1. Vandenbon A, Kumagai Y, Akira S, Standley DM. A novel unbiased measure for motif co-occurrence predicts combinatorial regulation of transcription. BMC genomics. 2012;13(Suppl 7):S11.

2. Hannenhalli S, Levy S. Predicting transcription factor synergism. Nucleic acids research. 2002;30(19):4278–4284.

3. Berman BP, Nibu Y, Pfeiffer BD, Tomancak P, Celniker SE, Levine M, et al. Exploiting transcription factor binding site clustering to identify cis-regulatory modules involved in pattern formation in the Drosophila genome. Proceedings of the National Academy of Sciences. 2002;99(2):757–762.

4. Lifanov AP, Makeev VJ, Nazina AG, Papatsenko DA. Homotypic regulatory clusters in Drosophila. Genome research. 2003;13(4):579–588.

5. Hu J, Lutz CS, Wilusz J, Tian B. Bioinformatic identification of candidate cis-regulatory elements involved in human mRNA polyadenylation. Rna. 2005;11(10):1485–1493.

6. Kiełbasa SM, Korbel JO, Beule D, Schuchhardt J, Herzel H. Combining frequency and positional information to predict transcription factor binding sites. Bioinformatics. 2001;17(11):1019–1026.

7. Wagner A. Genes regulated cooperatively by one or more transcription factors and their identification in whole eukaryotic genomes. Bioinformatics. 1999;15(10):776–784.
