# Supplementary figures and images for: Deciphering Cis-Regulatory Element Mediated Combinatorial Regulation in Rice under Blast Infected Condition
Source: PLoS One. 2015 Sep 1;10(9):e0137295. doi: 10.1371/journal.pone.0137295 (PMC4556519; doi:10.1371/journal.pone.0137295)

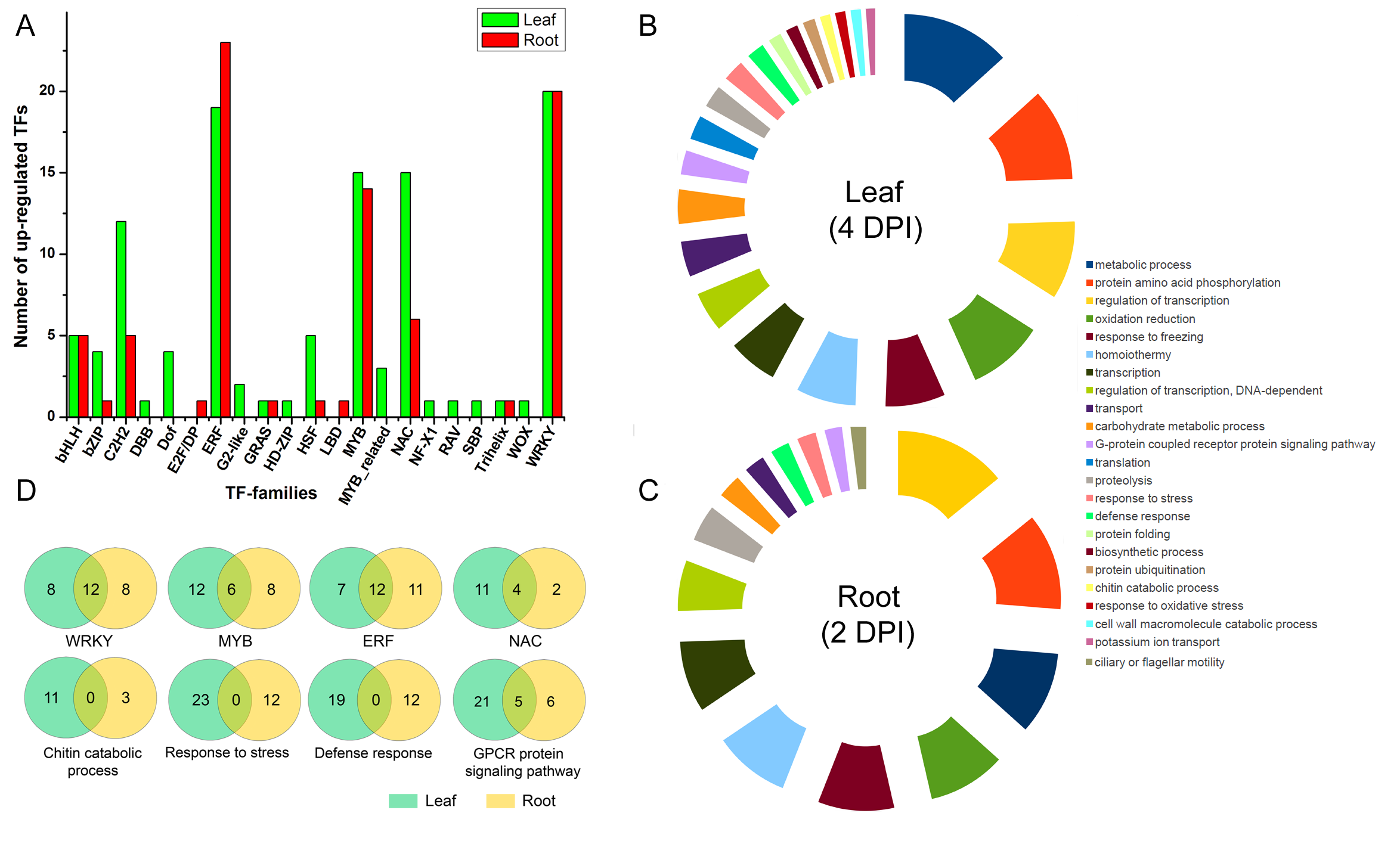

Supplement: S1 Fig — (A) Differentially up-regulated TFs in infected leaf and root of rice. The X axis represents individual TF-family and the Y axis represents the numbers of differentially up-regulated TF genes from each family. (B), (C) The pie charts representing the GO biological processes which mapped to ≥ 10 differentially up-regulated loci of infected rice leaf and root, respectively. In leaf 882 (out of 1201) loci and in root 502 (out of 677) loci are shown here. (D) The intersect region of each Venn diagram indicates the number of common up-regulated loci of respective gene-sets in between leaf (4 DPI) and root (2 DPI). (PNG) [file pone.0137295.s002.png]
